# Editorial: The pros and cons of psychotropic drug-induced changes in periphery and central nervous system: elucidating structural and molecular mechanisms

**DOI:** 10.3389/fpsyt.2023.1269307

**Published:** 2023-11-17

**Authors:** Roberto Esposito, HuaLin Cai, Wenbin Guo, Haibin Dai, Pei Jiang

**Affiliations:** ^1^Titano Diagnostic Clinic, Falciano, San Marino; ^2^Azienda Sanitaria Territoriale 1 (AST1), Pesaro-Urbino, Italy; ^3^Department of Pharmacy and Institute of Clinical Pharmacy, Second Xiangya Hospital of Central South University, Changsha, Hunan, China; ^4^Department of Psychiatry, National Clinical Research Center for Mental Disorders, and National Center for Mental Disorders, The Second Xiangya Hospital of Central South University, Changsha, Hunan, China; ^5^Second Affiliated Hospital, Zhejiang University School of Medicine, Hangzhou, China; ^6^Jining First People's Hospital, Jining, Shandong, China

**Keywords:** psychotropic drugs, brain structure abnormalities, cognitive function, peripheral metabolism, oxidative stress, genetic and environmental factors, neurotransmission, treatment response

## 1 Introduction

In recent years, psychotropic drugs have been widely prescribed in the treatment of neuropsychiatric diseases, contributing to various changes in the periphery and central nervous system (1). The regulation of psychotropic drugs is like a double-edged sword, not only manifested as therapeutic efficacy but also accompanied by adverse effects (2).

Advanced neuroimaging techniques represent a valid tool to study brain physiology and neural mechanisms in several pathological conditions such as neuropsychiatric disorders, allowing to examine brain structural and functional changes (3–5). Moreover, it represents a valid tool to understand the regulatory mechanisms of psychotropic drugs from the perspective of brain structure and function. Additionally, multiple factors have been proposed to account for the other biological changes in periphery and CNS induced by psychotropic drugs possibly through following mechanisms: (1) regulation of inflammation and oxidative stress signaling; (2) accumulation of advanced glycation end products (AGEs) occurred in carbonyl stress; (3) neurotransmission signaling and treatment response; (4) brain energy metabolism (e.g., mitochondrial function); (5) a combination of environmental factors (e.g., stress). In this Research Topic seven article were accepted after the review process regarding schizophrenia (SZ), antidepressant treatment (agomelatine), obsessive compulsive disorder (OCD) and sexual disfunction.

## 2 Schizophrenia

SZ is characterized by psychotic and negative symptoms and cognitive deficits with catastrophic effect on patients and their families. SZ may be considered a neurodevelopmental disorder.

Interesting result about SZ arise from the study of Jia et al. exploring influencing factors of cognitive impairments and their interrelationships in drug-naïve first-episode SZ. The authors analyze serum levels of oxidative stress indices (folate, superoxide dismutase, uric acid, homocysteine) correlating them to brain hippocampal volumes. The results show that oxidative stress impairs cognitive function by affecting hippocampal subfield volumes in early SZ.

A similar study was conducted by Wang et al. exploring the role of oxidative stress (blood serum glucose, superoxide dismutase, bilirubin) in first-episode SZ patients; they demonstrated an oxidative state imbalance in drug-naïve first-episode SZ patients, which might be associated with the pathogenesis of the disease adding that glucose, indirect bilirubin and superoxide dismutase may be potential biological markers of SZ.

An interesting point of view arises from the study of Li J. et al., that explores the role of microglia in SZ. Microglia could affect neuronal survival, neuronal death and synaptic plasticity during neurodevelopment and is involved in many neurodevelopmental diseases. Nowadays, accumulating experiments between microglia and SZ could afford unparalleled probability to assess this hypothesis.

Jiao et al. study treatment-resistant SZ patients, a clinical condition that often results in severe disability and functional impairment; the early identification of this category of patient allows the planning of specific therapeutic strategies to prevent a bad outcome. The predictors for treatment-resistant SZ remain to be explored even if peripheral biomarkers, show great potential which will enable individualized prediction and therapy for treatment-resistant SZ.

## 3 Obsessive-compulsive disorder

Chronic mental diseases such as OCD are associated with a high disability rate. Some patients still do not improve their symptoms even with adequate cognitive-behavioral therapy and drug treatment. Li K. et al. determines the efficacy electroconvulsive therapy in OCD. Electroconvulsive therapy is not considered a neuromodulation modality with sufficient evidence and its effectiveness is limited especially in patients with refractory symptoms.

## 4 Sexual disfunction

Sexual function is a complex behavior influenced by several factors and its disfunction is highly prevalent among patients with mental illness treated with psychotropic medications. Sewalem et al. in their cross-sectional study underlines the importance of enquire about sexual symptoms during follow-up treatment in order to give appropriate interventions to patients with chronic medical conditions and patients taking antipsychotics and psychotropic drugs.

## 5 Depression

Depression is a common emotional and mental disability characterized by the presence of depressed mood, the loss of interest or pleasure in daily activities, and other depression symptoms. It has a serious effect on the quality of life and increases their risk of developing physical and mental diseases.

Nesterowicz et al. studies agomelatine, an atypical antidepressant drug enhancing norepinephrine and dopamine liberation. Although this mechanism of action is the most studied, agomelatine moreover impacts on carbonyl/oxidative stress. Protein glycoxidation plays a crucial role in depression pathogenesis. In this study, molecular docking analysis of agomelatine in bovine serum albumin demonstrated its very low affinity could proclaim non-specific bonding and glycation factors attachment simplifying. Thereby the authors conclude that the drug may stimulate brain adaptation to carbonyl/oxidative stress.

## 6 Conclusions

Although results that arise from most recent studies regarding biomarkers related to psychotropic therapy remains fragmentary, when taken together, they can help to better understand the neurobiological bases (structural and molecular) of drug-induced changes in periphery and central nervous system. For example, molecular and genetic methods provide a means of predicting the molecular basis of treatment response. Furthermore, detection of peripheral immune system and stress biomarkers can be used to study the heterogeneity of psychotropic medication reactions. In conclusion, a combination of biomarkers could be a more effective strategy for predicting treatment outcomes. Some limitations are still present in this field of study: first of all, in many studies' periphery and central machanisms were usually considered separately. Future studies should consider high sample sizes and explore linking biomarkers that would reflect the mutual changes in the periphery and central nervous system.

## Author contributions

RE: Project administration, Supervision, Writing—original draft, Writing—review & editing. HC: Conceptualization, Data curation, Methodology, Project administration, Supervision, Writing—original draft, Writing—review & editing. WG: Conceptualization, Data curation, Methodology, Project administration, Resources, Software, Supervision, Validation, Visualization, Writing—original draft, Writing—review & editing. HD: Conceptualization, Data curation, Investigation, Project administration, Software, Supervision, Validation, Visualization, Writing—original draft, Writing—review & editing. PJ: Conceptualization, Data curation, Formal analysis, Funding acquisition, Investigation, Methodology, Project administration, Resources, Software, Supervision, Validation, Visualization, Writing—original draft, Writing—review & editing.
